# Study on lifestyle habits affecting sleep disorders at the undergraduate education stage in Xuzhou City, China

**DOI:** 10.3389/fpsyg.2022.1053798

**Published:** 2022-10-25

**Authors:** Qi Wu, Lei Yuan, Xiao-Han Guo, Jia-An Li, Dehui Yin

**Affiliations:** Key Laboratory of Human Genetics and Environmental Medicine, School of Public Health, Xuzhou Medical University, Xuzhou, China

**Keywords:** sleep disorders, physical exercise, undergraduate education, public health, cell phone use

## Abstract

**Background:**

In China, undergraduate students face both academic and career selection pressures, sleep is an important physiological process for them. Investigate the physical exercise, sleep quality of undergraduate students in the education stage in Xuzhou City, and analyze the factors affecting their sleep quality, to promote the health education and psychological health of undergraduate students.

**Materials and methods:**

The Physical Activity Rating Scale-3 (PARS-3), the Pittsburgh Sleep Quality Index (PSQI), and the demographic information questionnaire were used to survey a whole-group sample of four undergraduate colleges and universities (Xuzhou Institute of Engineering, Xuzhou Medical University, China University of Mining and Technology, Jiangsu Normal University) in Xuzhou by cluster sampling, the general characteristics including gender, grade, height, weight, domicile, race, economic income, etc., were collected, and the data were analyzed and processed using chi-square tests and multi-factor logistic regression.

**Results:**

3,366 valid questionnaires were collected from four undergraduate colleges and universities, including 1,355 males and 2,011 females. The detection rate of exercise in Jiangsu Normal University was lower than that in other universities, and the detection rate of sleep disorders was higher than that in other universities. Xuzhou Medical University, the highest detection rate of large exercise, Xuzhou Institute of Technology, the lowest detection rate of sleep disorders. There were significant differences in the detection rate of large amount of exercise among college students of different genders, grades, body types, and majors (χ^2^ = 259.172, *P* < 0.001; χ^2^ = 34.473, *P* < 0.001; χ^2^ = 36.026, *P* < 0.001; χ^2^ = 57.908, *P* < 0.001). There were significant differences in the detection rate of sleep disorders among college students with different gender, grade, family economic status, daily cell phone use time, cell phone purposeless usage, and exercise level (χ^2^ = 5.806, *P* = 0.016; χ^2^ = 47.5, *P* < 0.001; χ^2^ = 28.949, *P* < 0.001; χ^2^ = 55.866, *P* < 0.001; χ^2^ = 147.101, *P* < 0.001; χ^2^ = 9.129, *P* = 0.010). Multivariate logistic regression analysis showed that grade, family economic status, cell phone use time, cell phone purposeless usage is the main influencing factors of sleep disorders in college students.

**Conclusion:**

The sleep problems of undergraduates are serious, especially in Jiangsu Normal University. Scientific and appropriate exercise is an important measure to solve the sleep problems of undergraduates. Colleges and universities should actively carry out health education, college students living habits such as cell phone use should be guided training.

## Introduction

Sleep is an important physiological process for humans. During sleep, many important physiological changes occur in all body systems and organs, and once sleep deprivation occurs, physical and mental health problems follow (Chokroverty, 2010). Due to changing social pressures and increased academic demands, undergraduates often report poor sleep quality, which has become a global problem (Li et al., 2017). A mental health survey conducted by the WHO in 21 countries showed that 20.3% of college students suffer from mental health problems (Auerbach et al., 2016). In China, the number of undergraduate students in colleges and universities is huge, and this group faces both academic and career selection pressures. Research surveyed undergraduate students in 13 colleges and universities in China and found that, among them, 49.3% had a moderate degree of psychological stress, 8.4% had a critical mass with a heavy degree, and 0.3% had a very heavy degree of stress (Zhang et al., 2006).

Meanwhile, the incidence of sleep disorders in undergraduate student population is high due to their poor lifestyle habits (Falavigna et al., 2011). A survey in Guizhou, China, showed that about 53.7% of college students experienced poor sleep (Zhou et al., 2022). Sleep disorders among undergraduates have become a common and prominent harmful public health problem. Sleep loss may negatively impact on mood, cognitive and physiological functions, add risk of injury, etc., (Mah et al., 2018; Abdessalem et al., 2019; Li et al., 2020). There are many factors that affect the quality of sleep, including occupation, lifestyle, age, gender, etc., and lifestyle, such as cell phone use, physical exercise, has been shown to have a significant impact on sleep quality. The present study indicated that an active lifestyle could improve sleep quality significantly. Physical exercise not only affects sleep directly, but also influences one’s mental health status (Hee and Hyun, 2014). Studies show that exercise training increased sleep duration and variables of sleep quality in adolescents (Mendelson et al., 2016; Dolezal et al., 2017; Irandoust and Taheri, 2018). With the improvement of living standards, the use of cell phones is very common in the student population, almost all students have at least one cell phone, and the time and frequency of cell phone use is changing traditional lifestyles and habits. Especially since the outbreak of the COVID-19, people have reduced socialization and are facing more changing due to the need for epidemic prevention and control (Dergaa et al., 2021). Students have increased access to cell phone use and decreased physical activity, which may impair sleep and impact on individual quality of life, triggering various psychological problems such as depression and anxiety (Meng et al., 2021), which jeopardize the psychophysiological health of undergraduate students. Meanwhile, various factors such as psychological stress are positively associated with sleep deprivation (Wang et al., 2017), which may lead to more severe sleep difficulties. So, the necessity of conducting such research on the lifestyle habits affecting sleep disorders at the undergraduates is urgent.

Regarding to this issue, the purpose of this study is to investigate the physical exercise and sleep quality of undergraduates in different universities through detailed and extensive questionnaires, to analyze the relationship between exercise intensity and sleep quality of undergraduates in Xuzhou, to obtain the relevant factors affecting sleep quality. We hope these results can guide us to better understand the quality of sleep among undergraduates and what health-promoting activities can improve sleep quality. Informing health policymakers when it comes to improving the quality of sleep among college students.

## Materials and methods

### Data sources

From October to November 2019, undergraduates from four undergraduate universities in Xuzhou City (Xuzhou Institute of Engineering, Xuzhou Medical University, China University of Mining and Technology, Jiangsu Normal University) were selected as the subjects of cluster sampling survey. A total of 3,700 electronic questionnaires were distributed and collected in December 2019, and invalid questionnaires such as incomplete basic information and missing content were eliminated. A total of 3,366 valid questionnaires were recovered, with an effective recovery rate of 90.97%.

### Study methods

The questionnaire was used to investigate the basic situation of students’ physical exercise, sleep quality, and personal information. The main contents are as follows:

#### Physical activity rating scale-3 (PARS-3) (Yu et al., 2013)

According to the literature, PARS-3 is used to evaluate the amount of exercise of undergraduates. The scale quantifies the amount of exercise from the intensity, time, and frequency of participating in physical exercise. The amount of exercise = intensity × time × frequency. Intensity and frequency are divided into 1–5 levels, recorded as 1–5 points, time from 1 to 5 levels recorded as 0–4 points. Total score ≤19 is divided into small exercise, 20∼41 is divided into medium exercise, total score ≥42 is divided into large exercise.

#### Pittsburgh sleep quality index (PSQI) (Veqar and Hussain, 2020)

Pittsburgh sleep quality index was used to evaluate the sleep quality of the subjects in the past month and the items not included in the scale were deleted. The remaining 18 items of self-assessment consisted of seven components (subjective sleep quality, sleep latency, sleep time, sleep efficiency, sleep disorders, hypnotic drugs, and daytime dysfunction). Each component was scored by 0–3 points, and the total score ranged from 0 to 21 points. The higher the score, the worse the sleep quality. If the score was ≥8 points, there was sleep problem. The Cronbach’s alpha coefficient is 0.835, and the Kaiser-Meyer-Olkin value is 0.890 (*P* < 0.001). Scores of the reliability and validity indicated the questionnaire is highly acceptable.

To increase some demographic information and other survey items of students according to the purpose of the study. Independent increase projects include gender, grade, height, weight, household registration, ethnicity, income, and college students to use cell phone survey. Obesity was evaluated by body mass index (BMI) in the classification standard of body mass index for overweight and obesity screening in Chinese school-age children and adolescents. BMI < 18.5 was thin, 18.5∼23.9 was normal, 24.0∼26.9 was fat, and BMI ≥ 27.0 was obese (Cao et al., 2022).

### Statistical analysis

GraphPad Prism software (version 6.05) and SPSS25.0 statistical software was used to rationalize the differences in exercise intensity, sleep quality, and anxiety status of different demographic indicators. Qualitative data were expressed as rate (%) or composition ratio (%). Chi-square (χ^2^) test was used for single factor analysis. Logistics multivariate regression analysis was used to analyze the factors affecting sleep quality, and the test level was α = 0.05. Data management and analysis was conducted in December 2019.

## Results

### General characteristics of participants

In this survey, 3,366 valid questionnaires were collected, including 1,355 boys and 2,011 girls. The basic information includes grade, family economic status, native place, major and so on (Table 1).

**TABLE 1:** Basic characteristics of participants.

Group		Number	Rate (%)	Group		Number	Rate (%)
Gender	Male	1,355	40.3	Major	Literature	1,208	35.9
	Female	2,011	59.7		Science	538	16
Grade	First year	1,762	52.3		Engineering	1,008	29.9
	Second year	1,098	32.6		Medicine	612	18.2
	Third year or more	506	15.1	Body type	Obesity	283	8.4
Family economic status	Poor	1,017	30.2		Fat	340	10.1
	Medium	2,261	67.2		Thin	600	17.8
	Good	88	2.6		Normal	2,143	63.7
Native place	Urban	1,396	41.5	Cell phone purposeless use	Never	610	18.1
	Rural	1,970	58.5		Occasional	1,517	52.2
Daily cell phone use time	1∼4 h	1,269	37.7		Often	999	29.7
	5∼8 h	1,615	48	Watching cell phone before bed			
	9∼12 h	343	10.2		Yes	3,211	95.4
	12 h or more	139	4.1		No	155	4.6

### Physical activity of the survey respondents

**FIGURE 1:**
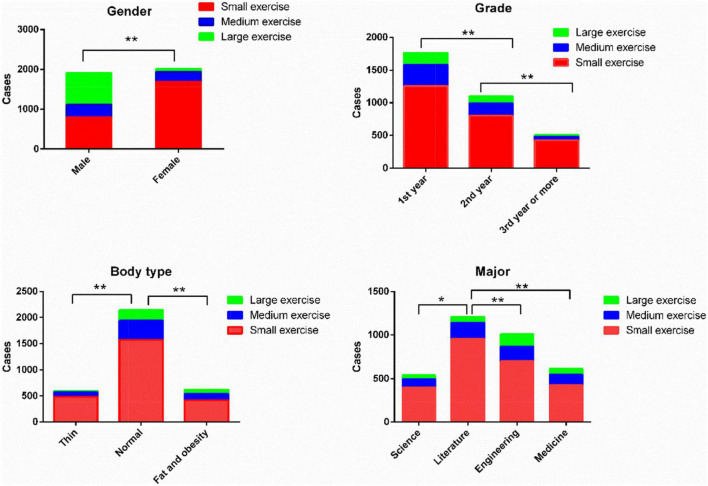
Physical activity of college students. *P < 0.05, **P < 0.01.

The results of the study showed that among 3,366 college students, the proportion of male students participating in medium and large exercise (41.03%) was higher than the proportion of female students participating in medium and large exercise (16.16%) (χ^2^ = 259.172, *P* < 0.01); the proportion of third year or more students participating in medium and large exercise (15.8%) was lower than that of first year students (28.7%) (χ^2^ = 33.987, *P* < 0.01) and second year (26.9%) (χ^2^ = 23.897, *P* < 0.01); the proportion of obese and obese students participating in medium and large exercise (32.6%) was higher than that of normal-sized students (26.5%) (χ^2^ = 9.017, *P* < 0.05) and thin students (18.5%) (χ^2^ = 15.954, *P* < 0.01); there was no statistical difference between the proportion of urban students participating in medium and large exercise and the proportion of rural students participating in medium and large exercise; the proportion of students in literature majors participating in medium and large exercise (20.7%) was lower than that of students in science (25.7%), engineering (30.7%), and medicine (30.1%) (χ^2^ was 5.288, 28.895, and 19.639, respectively; *P* < 0.05, <0.01, and <0.01, respectively). Detailed results are shown in Table 2 and Figure 1.

**TABLE 2:** Physical activity of college students.

Group		Small exercise (%)	Medium exercise (%)	Large exercise (%)	χ^2^
Gender	Male	799 (59.0	313 (23.1)	243 (17.9)	298.124**
	Female	1,686 (83.8)	252 (12.5)	73 (3.6)	
Grade	First year	1,257 (71.3)	324 (18.4)	181 (10.3)	34.473**
	Second year	802 (73.0)	187 (17.0)	109 (9.9)	
	Third year or more	426 (84.2)	54 (10.7)	26 (5.1)	
Body type	Thin	489 (81.5)	80 (13.3)	31 (5.2)	36.026**
	Normal	1,576 (73.5)	364 (17.0)	203 (9.5)	
	Fat and obesity	420 (67.4)	121 (19.4)	82 (13.2)	
Major	Literature	958 (79.3)	183 (15.1)	67 (5.5)	57.908**
	Science	400 (74.3)	96 (17.8)	42 (7.8)	
	Engineering	699 (69.3)	168 (16.7)	141 (14.0)	
	Medicine	428 (69.9)	118 (19.3)	66 (10.8)	

**P < 0.01.

### Comparison of survey items among the four universities

The reported rate of medium to large exercise among students in Jiangsu Normal University was 17.9%, which was the lowest among the four universities, smaller than that of China University of Mining and Technology (26.6%, χ^2^ = 14.776, *P* < 0.01), Xuzhou Engineering College (29.1%, χ^2^ = 30.361, *P* < 0.01), Xuzhou Medical University (29.7%, χ^2^ = 33.877, *P* < 0.01); the lowest detection rate of sleep disorders was 12.4% in Xuzhou Engineering College, which was smaller than the detection rate in China University of Mining and Technology (16.7%, χ^2^ = 5.503, *P* < 0.05), Xuzhou Medical University (16.8%, χ^2^ = 7.631, *P* < 0.01), and Jiangsu Normal University (17.7%, χ^2^ = 9.841, *P* < 0.01); there was no statistical difference in sleep time among college students, and the overall proportion of sleep time was 44.9% for 6∼7 h, 41.0% for more than 7 h, and 14.2% for less than 6 h; the proportion of looking at cell phone before bedtime was 95.4%; the proportion of daily cell phone use time was 37.7% for 1∼4 h, 48.0% for 5∼8 h, and 14.3% for more than 9 h. The detailed results are shown in Table 3.

**TABLE 3:** Comparison of survey items among four universities.

Survey items		XZMU (%)	CUMT (%)	XZNU (%)	XZIT (%)
Medium to large exercise reporting rate		29.7	26.6	17.9	29.1
Sleep disorder detection rate		16.8	16.7	17.7	12.4
Daily sleep time	7 h or more	40.0	33.8	37.7	48.8
	6∼7 h	46.5	52.9	46.3	37.4
	6 h or less	13.5	13.3	16.0	13.7
Watching cell phone before bed		96.2	96.2	93.7	95.4
Cell phone purposeless usage	Occasional	68.7	65.3	69.2	75.8
	Often	31.3	34.7	30.8	24.2
Daily cell phone use time	1∼4 h	43.4	33.6	37.0	34.7
	5∼8 h	42.8	52.2	51.1	48.3
	9 h or more	13.7	14.2	11.9	17.0

### Comparison of the detection rate of sleep disorders among different categories of college students

The total detection rate of sleep disorders was 15.7% in all surveys. The detection rate of sleep disorders in male students (13.9%) was lower than that in female students (17.0%) (χ^2^ = 5.806, *P* < 0.05); the detection rate of sleep disorders in obese students was 14.9%, in normal-sized students, it was 15.5%, and in thin students, it was 17.2%, with no statistically significant difference (χ^2^ = 1.296, *P* > 0.05); the detection rate of sleep disorders among second year students (17.9%) was greater than that of first year students (12.0%) (χ^2^ = 18.727, *P* < 0.01), and the detection rate of sleep disorders among third year or more students (23.9%) was higher than that of second year students (17.9%) (χ^2^ = 8.028, *P* < 0.01). The difference was not statistically significant; the detection rate of sleep disorders was 15.4% for students from urban areas and 15.9% for students from rural areas; the detection rate of sleep disorders among students with poor family economic status (20.8%) was higher than that of students with good family economic status (13.5%) (χ^2^ = 28.949, *P* < 0.01); the detection rate of sleep disorders among literature majors was 16.9%. The detection rate of sleep disorders was 16.1% for literature majors, 18.4% for science majors, 13.6% for engineering majors, and 16.2% for medical majors, and the differences were not statistically significant in both comparisons. The detailed results are shown in Table 4.

**TABLE 4:** Comparison of the detection rate of sleep disorders among students in different groups of universities.

Group		Sleep disorder	χ^2^
Gender	Male	188 (13.9)	5.806*
	Female	341 (17.0)	
Grade	First year	212 (12.0)	47.5**
	Second year	196 (17.9)	
	Third year or more	121 (23.9)	
Native place	Urban	215 (15.4)	0.178
	Rural	314 (15.9)	
Family economic status	Poor	212 (20.8)	28.949**
	Medium or more	317 (13.5)	
Major	Literature	194 (16.1)	6.570*
	Science	99 (18.4)	
	Engineering	137 (13.6)	
	Medicine	99 (16.2)	
Cell phone use time	1∼4 h	141 (11.1)	55.866**
	5∼8 h	265 (16.4)	
	9 h or more	123 (25.5)	
Cell phone purposeless usage	Occasional	255 (10.8)	147.101**
	Often	274 (27.4)	
Exercise level	Small	418 (16.8)	9.129**
	Medium	68 (12.0)	
	Large	43 (13.6)	

*P < 0.05, **P < 0.01.

### Multi-factor logistic regression analysis of sleep quality status

**FIGURE 2:**
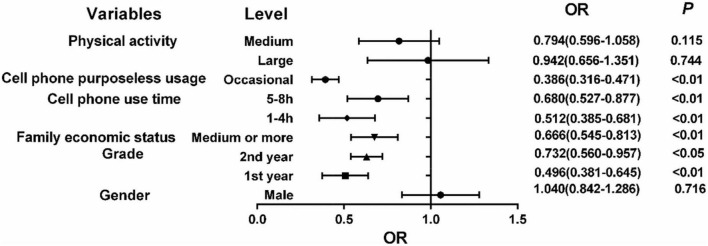
Results of multi-factor logistic regression analysis of sleep quality status.

With the presence of sleep disorder as the dependent variable (0 = no, 1 = yes), the independent variables gender (0 = male, 1 = female), grade (1 = first year, 2 = second year, 3 = third year or more), family economic status (0 = medium and above, 1 = poor), major (1 = literature, 2 = science, 3 = engineering, 4 = medicine) daily cell phone use time (1 = 1∼4 h, 2 = 5∼8 h, 3 = 9 h, or more), cell phone purposeless usage (0 = occasional, 1 = often), and exercise level (1 = large exercise, 2 = medium exercise, 3 = small exercise) were entered into logistic regression model analysis, and the analysis results showed that grade, family economic status, cell phone usage time, and purposeless cell phone viewing behavior were the main factors generating sleep disorders among college students. The detailed results are shown in Table 5 and Figure 2.

**TABLE 5:** Results of multi-factor logistic regression analysis of sleep quality status.

Variables		β	SE	Wald	OR (95% CI)	*P*
Gender	Male	0.039	0.108	0.133	1.040 (0.842–1.286)	0.716
Grade	First year	–0.702	0.134	27.452	0.496 (0.381–0.645)	< 0.01
	Second year	–0.311	0.137	5.190	0.732 (0.560–0.957)	< 0.05
Family economic status	Medium or more	–0.407	0.102	15.854	0.666 (0.545–0.813)	< 0.01
Cell phone use time	1∼4 h	–0.670	0.145	21.195	0.512 (0.385–0.681)	< 0.01
	5∼8 h	–0.385	0.130	8.815	0.680 (0.527–0.877)	< 0.01
Cell phone purposeless usage	Occasional	–0.953	0.102	87.281	0.386 (0.316–0.471)	< 0.01
Exercise level	Large	–0.060	0.184	0.107	0.942 (0.656–1.351)	0.744
	Medium	–0.231	0.146	2.480	0.794 (0.596–1.058)	0.115
Constant		0.414	0.288	2.069	1.513	0.150

## Discussion

Sleep problems are related to whether students can have sufficient energy for a day of study and life, and affect the quality of learning. However, due to the large academic load of undergraduates, the huge employment pressure they face, and the current popularity of smart phones and other devices, the division de current undergraduates have serious sleep problems, and finding out the factors that affect the sleep quality of undergraduates is essential for preventive interventions in undergraduates, in which health education is carried out to improve the quality of students’ sleep is crucial.

The results of this study show that Jiangsu Normal University stands out from other schools in terms of both exercise and sleep problems, which may be related to the composition of the liberal arts majority in Jiangsu Normal University and the learning and living environment; some studies have shown that medical students are more likely to be affected by sleep disorders than students of other majors, which is generally considered to be caused by the increased psychological stress and anxiety due to the large study load of medical students (Azad et al., 2015). In contrast, this study showed that the detection rate of sleep disorders in Xuzhou Medical University was lower than the other three universities, which may be related to the professional knowledge and stronger health concept possessed by medical students, which prompted them to exercise consciously; the lowest sleep quality detection in Xuzhou Engineering College may be related to the professional learning characteristics of engineering colleges that focus on practice, in which students can exercise better, and the practical courses require more participation of students with certain hands-on ability, etc., which are more popular among students compared to the boring classroom teaching. Therefore, the proportion of practical courses should be increased, diversified teaching should be promoted, and the reform of related teaching courses should be strengthened in the undergraduate education stage of colleges and universities.

The results of this study showed that male students exercised significantly more than female students in four colleges and universities in Xuzhou, which was consistent with previous studies. Among different grades, the higher the grade, the lower the amount of exercise, and the decline in the level of exercise among senior students is prominent, which may be related to the gradual increase in the curriculum of senior students in the later years, and the fact that senior students face graduation and planning for their future careers or studies, and can spend less time on physical exercise. Therefore, higher education institutions should coordinate and balance the number of courses for each grade level when setting the curriculum. The low exercise level of low weight college students and the higher exercise level of obese students may be related to the fact that obese students have the desire to lose weight and have stronger motivation to exercise. The current study showed that the detection rate of sleep disorders among college students was 15.7%, which is lower than the 25.7% detection rate of sleep disorders among college students from a study involving 1,12,939 students (Li et al., 2018), which may be related to the regional differences between the study and living environment of the college student population in this study and other studies, so that the scientific health education should be carried out according to local conditions and the relevant departments of the school, taking into account the specific conditions of their own schools. The detection rate of sleep disorders in senior students is higher than that of junior students, probably due to the fact that senior students face problems of graduation and career selection making psychological pressure higher, which affects the quality of sleep. This study confirms that family economic situation affects students’ sleep, and students with poorer family economic situation have more serious sleep disorders, probably due to the fact that students with poorer economic situation have more stressful life and are more likely to have anxiety and affect the quality of sleep. The higher the frequency of purposelessly looking at cell phones and the longer the daily time spent looking at cell phones in this study, the higher the detection rate of sleep disorders, which is consistent with the results of domestic and international studies (Monma et al., 2018; Feng et al., 2022), probably due to the fact that anxious students are more inclined to use cell phones to escape from difficulties to relieve negative emotions, which also affects the sleep time and sleep quality and leads to sleep deprivation. In colleges and universities, it has been the norm for undergraduates to have a cell phone in their hands, and students often play with their cell phones in class, which not only affects students’ learning effect but also makes myopia more and more serious among undergraduates nowadays, therefore, colleges and universities should restrict students from playing with their cell phones in class, and relevant departments of schools, such as academic affairs office and student work office, should strengthen the supervision of students and make corresponding system documents to restrict cell phones in class. At the same time, they should strengthen publicity and education to make students clear the harm of looking at cell phones for a long time. At present, many colleges and universities, including Xuzhou Medical University, have prepared storage bags for cell phones inside classrooms, but the utilization rate is low, and few students take the initiative to put their cell phones into the bags.

The Logistic regression analysis showed that physical exercise was not a factor affecting sleep quality, while univariate analysis showed that there were differences in sleep quality between groups in terms of the amount of exercise. Another study confirmed that physical exercise influenced sleep quality, and that people with medium exercise had better sleep quality at different exercise intensities (Ji et al., 2022). Study has confirmed that an active lifestyle, especially with moderate physical activity, can improve the quality of sleep (Taheri and Irandoust, 2020). Physical activity can promote sleep by changing the composition of the body including plasma concentrations of catecholamines, cortisol and melatonin, rectal temperature, polysomnography, heart rate variability, and stimulating the sympathetic nervous system (Yamanaka et al., 2015; Paryab et al., 2021). Studies also indicated that physical activity could decrease NREM stage N1 (very light sleep) while increasing REM sleep, sleep continuity, and sleep efficiency (Mendelson et al., 2016). Therefore, physical exercise should be enhanced among students in the college population. Research also confirmed that medium physical exercise can improve students’ anxiety and relieve psychological burden, which in turn can affect sleep quality (Yin et al., 2022). Therefore, whether physical exercise is an influencing factor of sleep disorder or not needs further study.

Logistics regression analysis shows that grade level, family economic status, cell phone use time, and cell phone purposeless use are the main influencing factors of college students’ sleep disorder, therefore, schools should target different forms of publicity and education in different grades; selectively intervene in the high-risk student population by combining students’ family economic status; they should also care more about the poorer family economic status and pay more attention to students from poorer families to reduce their psychological burden. On the issue of students looking at cell phones, schools should adopt a combination of education and management, advocating students to reduce the time they spend looking at cell phones on their own initiative while adopting some systems to reduce the time students spend looking at cell phones through supervision and management.

In summary, according to the results of this study, the following measures should be taken in order to improve the sleep quality of undergraduate students: for colleges and universities where students lack medical knowledge, have weak health concepts and have a heavy academic burden (such as teacher training colleges), health education should be focused and lectures should be organized so that non-medical students can fully understand sleep disorders, develop good health habits and improve the quality of sleep. Moderate exercise can both reduce anxiety levels and improve sleep quality, and there is a strong correlation between anxiety levels and sleep disorders, and a reduction in anxiety levels also directly affects sleep, so scientific and moderate exercise is the key initiative to solve the anxiety and sleep problems of college students. It is necessary to actively participate in physical exercise especially for senior students. It is also necessary to guide and cultivate the habits of college students, such as mobile phone use, and allocate the time after school to colorful practical activities.

### Limitations

Currently, the factors affecting sleep have been confirmed to include psychological, physiological, nutrition, etc. However, this study is a simple cross-sectional study that only analyzed the effect of lifestyle habits on sleep, and although some influencing factors regarding the impact of sleep were derived, no in-depth mechanism exploration was conducted, and these factors need to be corroborated by cohort or experimental epidemiology studies.

## Data availability statement

The original contributions presented in this study are included in the article/supplementary material, further inquiries can be directed to the corresponding author.

## Author contributions

DY conceived and designed the study. X-HG, QW, and LY analyzed the data. J-AL drafted the manuscript. DY reviewed and made improvements in the manuscript. All authors read and approved the final manuscript.
